# From Polygenic Scores to Precision Medicine in Alzheimer’s Disease: A Systematic Review

**DOI:** 10.3233/JAD-191233

**Published:** 2020-04-21

**Authors:** Judith R. Harrison, Sumit Mistry, Natalie Muskett, Valentina Escott-Price

**Affiliations:** a Cardiff University Brain Research Imaging Centre (CUBRIC), Cardiff, UK; b MRC Centre for Neuropsychiatric Genetics and Genomics, Hadyn Ellis Building, Cardiff University, Cardiff, UK; c Cardiff University Medical School, University Hospital of Wales, Cardiff, UK; d Dementia Research Institute & the MRC Centre for Neuropsychiatric Genetics and Genomics, Hadyn Ellis Building, Cardiff University, Cardiff, UK

**Keywords:** alleles, Alzheimer’s disease, amyloid-beta peptides, cognitive dysfunction, genome-wide association study, multifactorial inheritance, neuroimaging, phenotype, precision medicine, single nucleotide polymorphism

## Abstract

**Background:**

Late-onset Alzheimer’s disease (AD) is highly heritable. The effect of many common genetic variants, single nucleotide polymorphisms (SNPs), confer risk. Variants are clustered in areas of biology, notably immunity and inflammation, cholesterol metabolism, endocytosis, and ubiquitination. Polygenic scores (PRS), which weight the sum of an individual’s risk alleles, have been used to draw inferences about the pathological processes underpinning AD.

**Objective:**

This paper aims to systematically review how AD PRS are being used to study a range of outcomes and phenotypes related to neurodegeneration.

**Methods:**

We searched the literature from July 2008–July 2018 following PRISMA guidelines.

**Results:**

57 studies met criteria. The AD PRS can distinguish AD cases from controls. The ability of AD PRS to predict conversion from mild cognitive impairment (MCI) to AD was less clear. There was strong evidence of association between AD PRS and cognitive impairment. AD PRS were correlated with a number of biological phenotypes associated with AD pathology, such as neuroimaging changes and amyloid and tau measures. Pathway-specific polygenic scores were also associated with AD-related biologically relevant phenotypes.

**Conclusion:**

PRS can predict AD effectively and are associated with cognitive impairment. There is also evidence of association between AD PRS and other phenotypes relevant to neurodegeneration. The associations between pathway specific polygenic scores and phenotypic changes may allow us to define the biology of the disease in individuals and indicate who may benefit from specific treatments. Longitudinal cohort studies are required to test the ability of PGS to delineate pathway-specific disease activity.

## INTRODUCTION

Alzheimer’s disease (AD) is a common neurodegenerative condition affecting people in later life. The heritability of late-onset AD is estimated to be almost 75% [1]. Genome-wide association studies (GWAS) have identified a number of loci associated with AD. The largest meta-analysis to date reported 25 loci associated with increased risk for AD at genome-wide significant level [2]. These common genetic variants, known as single nucleotide polymorphisms (SNPs), have only a small effect on disease risk.

Polygenic risk scores (PRS) sum the weighted allelic dosages across the genome, and have allowed the exploration of how genetic risk for AD is manifest in different populations [3]. However, genetic score methodology varies greatly between studies. For example, Escott-Price et al. analyzed over 200,000 SNPs, including *APOE* and reported an area under the curve (AUC) value of 0.84 [4] whereas Tosto et al. used only 21 SNPs excluding APOE resulting in an AUC of 0.57 [5].

As GWAS allows all variants in the genome to be tested for association simultaneously without any *a priori* hypothesis, they have implicated a number of areas of biology previously unconnected to AD. Pathway analyses of genome-wide association data have shown that the disease processes that underpin AD are highly complex, involving a number of biological processes, including immunity, lipid metabolism, tau binding proteins, and amyloid-β protein precursor metabolism [2, 6].

Since the PRS approach was first described, many studies have investigated whether AD PRS are associated with a wide variety of phenotypes. To summarize this literature, we undertook a systematic review to identify studies that have used a PRS approach to investigate phenotypes associated with genetic risk for AD.

## METHODS

The review was conducted in accordance with the PRISMA guidelines for systematic reviews [7]. (please see PRISMA checklist in Supplementary Materials).

### Search strategy

We searched MEDLINE, PSYCHINFO, and EMBASE literature from July 2008–July 2018. We used a list of predetermined search terms listed in Supplementary Table 1, and also manually searched the reference lists of relevant articles.

#### Inclusion criteria:

• Longitudinal, cross-sectional or case-control studies including genotyped data;
• Validated risk loci for AD identified and combined into a PRS;
• Reported associations with AD case/control status or another phenotype.

#### Exclusion criteria:

• Studies reporting associations with family history only;
• Studies reporting on genetic risk for other conditions or loci that have not been previously shown to increase risk of AD;
• Studies reporting the effect of only one locus or gene (e.g., *APOE*), or *APOE* combined with non-genetic risk factors;
• Non-English publications in the absence of resources to translate, or an existing translation.

### Article selection

All articles selected for inclusion were original research reports written in English. The design of the studies was cross-sectional, longitudinal or observational. The initial search was conducted by NM. Based on the eligibility criteria, two reviewers (JH and SM) independently selected studies. Any discrepancies were resolved by a third reviewer (VEP).

### Data extraction

The reviewers (JH and SM) extracted data from the studies independently and in duplicate. The extracts included: 1) the type of study, 2) the discovery sample (study name, sample size and number of cases), 3) the target sample (study name, sample size, and case number); and 4) the number of SNPs included in the PRS (see data extraction form in Supplementary Material). Results that were reported in separate papers were only included once.

## RESULTS

### Search results

**Fig.1:**
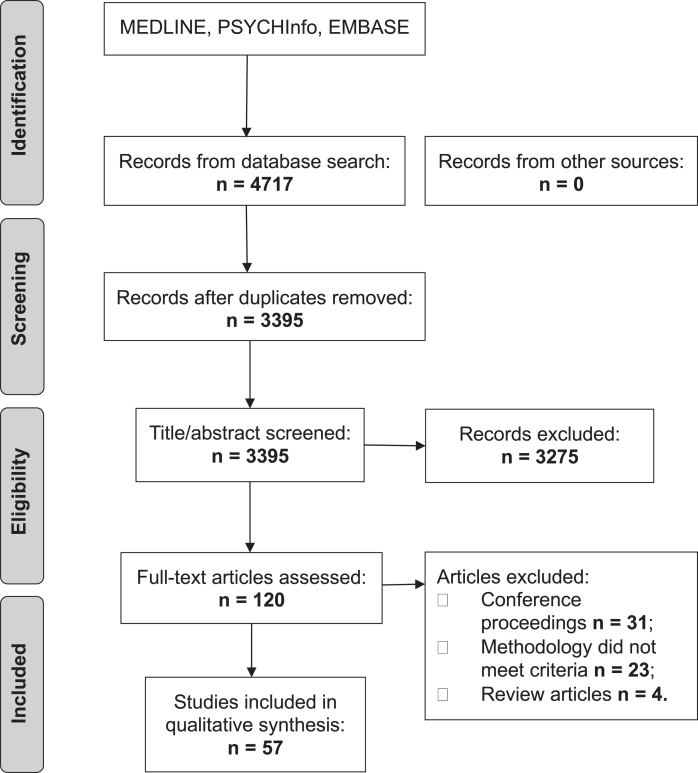
PRISMA flow chart.

The initial search produced 4,717 articles (see PRISMA flow chart in Fig. 1). 1,322 were removed as duplicates. A further 3,275 were excluded based on their title and abstract. The reviewers (JH and SM) reviewed the full text of the remaining 120 articles and applied strict inclusion criteria, excluding a further 63. 57 articles were eligible for inclusion.

There was only one disagreement between raters which was resolved by a third reviewer (VEP).

### Study characteristics

There was a variety of study designs. Most were case-control studies, comparing those with AD or mild cognitive impairment (MCI) to healthy controls [4, 5, 8–29]. Others were cross-sectional [20, 21, 30–58] and some were longitudinal [59–64]. The majority included participants of European ancestry from Europe, the US or Australia, although some included Black African American [39, 44, 45], Hispanic [5, 40], Caribbean [5], or Han Chinese participants [13, 22, 57]. Sample size ranged from 66 [65] to over one hundred thousand [47]. The articles examined associations with several phenotypes. See Table 1 for a summary of study characteristics.

**Table 1:** Summary of included studies by type of PRS

Correlates/Outcomes	N Studies	N Threshold PRS Studies	N GWAS Significant PRS Studies
AD risk prediction	15	5	10
MCI risk prediction or MCI conversion	4	2	2
MRI phenotypes	12	7	5
Cognition	21	5	16
CSF biomarkers	8	3	5
Other diseases/syndromes	4	2	2
Disease pathways	3	1	2

AD, Alzheimer’s disease; MCI, mild cognitive impairment; MRI, magnetic resonance imaging; CSF, cerebrospinal fluid; GWAS, genome-wide association study.

### PRS calculation

All studies computed PRS using SNPs that have been associated with AD in large meta-analyses. Most used the International Genomics of Alzheimer’s Project (IGAP) [66] or another recent GWAS. There were two approaches to identifying SNPs for inclusion: 1) selecting SNPs that reached genome-wide significance in meta-analysis, or 2) using *p*-value thresholds, including a greater number of nominally associated SNPs (please see Supplementary Tables 3 and 4 for a summary of PRS calculation). The number of studies using each approach is outlined in Table 1. Two of the studies in Han Chinese populations choose to verify that the SNPs were associated with AD in their population before computing PRS [13, 22]. Most studies weighted PRS by effect size, specifically the logarithm of the odds ratio or beta-coefficient from the regression analysis model, as described by Purcell and colleagues [67]. There were five exceptions: one study weighted by explained variance [37]; four studies created unweighted scores by summing the number of risk loci [27, 39, 52, 68]. *APOE* was either included as a co-variate, included in the PRS or excluded (see Supplementary Tables 3 and 4).

### Prediction of AD case/control status

15 studies used PRS to predict AD case/control status with various statistical approaches. Some studies used the area under the receiver operating characteristic (ROC) curve, whereas others used time-to-event analysis, odds ratios (OR), or a combination of methods. All found that PRS was able to discriminate cases from controls, although prediction accuracy varied.

Of those studies reporting area under the curve (AUC), five included *APOE* and achieved AUC ranging from 0.62–0.84 [12, 15–17, 22, 69]. Four studies excluded *APOE* and achieved AUC ranging from 0.57–0.75 [5, 12, 25, 30]. Of those studies using time-to-event analysis, all four excluded APOE and reported hazard ratios (HR) ranging from 1.11–2.36 [23, 35, 70]. Of those studies using ORs, two included *APOE* in their PRS and reported OR ranging from 2.06–2.32 [12, 34]. Four studies excluded *APOE* and reported OR ranging from 1.14–2.85 [5, 25, 30, 71]. For more detailed information including the details of the samples and outcome measures used by each study, please see Supplementary Tables 3 and 4.

### Mild cognitive impairment to AD conversion

Eight studies assessed the ability of PRS to predict MCI to AD conversion. Three studies did not report statistically significant results [11, 29, 37]. Rodriguez-Rodriguez et al. compared those in the 1^st^ and 3^rd^ tertile of PRS (OR: 1.32, 95% CI: 0.57–3.06). Neither of the hazard models used by Lacour et al. and Andrews et al. produced significant results (Lacour HR: 1.18, 95% CI: 0.37–2.0; Andrews HR: 1.05, 95% CI: 0.86–1.29) [29, 37]. However, Andrews et al found their PRS was associated with an increased risk of transitioning from normal cognition to dementia (HR = 4.19, 95% CI: 1.72–10.20) [37]. Five studies did report statistically significant results [18, 33, 35, 58, 64]. However, when *APOE* was removed, only one study remained positive [58]. An additional study evaluated genetic contributors to the Diagnostic and Statistical Manual IV (DSM-IV) diagnosis of Cognitive Impairment, No Dementia, which is similar to MCI. They found no significant difference in the frequency of risk alleles between cases and controls (*p* = 0.710) [27].

### Cognitive measures

Cognition and PRS were examined in 21 studies [11, 26, 31, 33, 36, 39, 40, 44, 45, 50–52, 54, 56, 57, 59, 60, 62–64, 68]. While a variety of cognitive measures were used, all but four studies reported some significant associations with PRS. Most studies were in healthy older adults, although two studies included participants with established AD/MCI [11, 26], two studies had young adult participants [51, 57], one study had adolescent participants [40] and one included longitudinal data from children aged 11 [59]. There were some cross-sectional studies that only reported associations with AD polygenic risk and cognition at one timepoint [40, 50–52, 54, 55, 57, 68], whereas longitudinal studies were able to report the correlations with change in cognition over time [11, 26, 31, 33, 36, 44, 45, 56, 60, 62–64]. As expected, most studies reported that the effects attenuated or were no longer significant when *APOE* was excluded from the PRS. Please see Supplementary Tables 3 and 4 for full details of cohorts and measures used.

### MRI phenotypes

12 studies explored correlations between AD PRS and MRI phenotypes. Most studies looked at subcortical volumes [13, 30, 33, 40, 51]. Some also explored cortical thickness [34, 41, 56], white matter metrics [51], and functional MRI [13]. One study used algorithms that assess spatial atrophy patterns in AD [43]. Most studies sampled healthy older adults, although some included younger adults [51, 57], adolescents [40], or a range of age groups [30]. Some studies included some participants with MCI or AD [13, 30, 33] and one study sampled military veterans with head injuries [56].

Of the six studies that explored subcortical volumes, all reported significant negative correlations between PRS and hippocampal volume [30, 33, 40, 51, 61, 72]. Hohman et al. only found a significant association in participants who were negative for amyloid on PET [28]. Lupton et al. reported a significant negative association with amygdala volume [30] but only in participants with diagnoses of MCI or AD. Habes et al. trained an algorithm to detect the spatial patterns of healthy brain aging and atrophy in AD. They found a significant association AD PRS and the spatial pattern AD atrophy [43].

Of those studies looking at cortical thickness [34, 41, 56, 57, 61], all but one [61] reported significant associations between increased PRS and cortical thinning. Studies either reported associations with cortical thinning across multiple regions that are susceptible to AD pathology [34, 41, 56], or with cortical thinning is specific regions such as the precuneus [57].

Foley et al. assessed white matter, and identified reduced fractional anisotropy in the right cingulum with increasing PRS [51]. Su et al. explored changes in the default mode network and reported changes in functional connectivity in the left medial temporal gyrus and the right hippocampal/parahippocampal gyrus in those with MCI. However, there were no significant associations in healthy controls [13].

### Amyloid and tau biomarkers

Nine studies explored associations between PRS and amyloid and tau biomarkers [8–10, 21, 25, 33, 35, 49, 60]. They were all case/control studies. One study sampled those with autosomal dominant and sporadic AD [25]. Another included participants with normal pressure hydrocephalus [8]. The phenotypes included: cerebrospinal fluid (CSF) amyloid and tau measures [10, 21, 25, 33, 35, 49]; postmortem biomarkers or histology [8, 10, 35]; amyloid PET [33, 35].

A variety of analysis approaches were taken. Some studies assessed each tau and amyloid biomarker independently [9, 10], whereas others created composite variables using CSF, PET, or histology biomarkers [21, 25, 35, 49].

There were significant associations reported between AD PRS and the following: increased CSF tau and phosphorylated tau [9]; CSF Aβ [10]; lower Aβ_42_/Aβ_40_ [49]; higher t-tau/Aβ_42_ and higher p-tau/Aβ_42_ ratio [25, 49]; positive Aβ PET [33]; total PET/CSF amyloid load and tau load [35]; postmortem soluble Aβ_42_ and *λ*-secretase activity [10]; postmortem amyloid plaques and neurofibrillary tangles [60]. Some studies did not report significant associations between AD PRS and CSF tau [10, 33] or CSF Aβ [9, 33]. There was also no association with microglial density on postmortem histology [60] or amyloid deposition in brain biopsies of normal pressure hydrocephalus patients [8]. Voyle et al. combined CSF biomarkers with PRS to predict AD, but the PRS did not improve prediction over and above the CSF amyloid and tau [21].

### Other diseases and syndromes

Other studies have explored associations between AD PRS and other disorders or syndromes. Pilling and colleagues reported significant negative correlations with longevity [47], and red cell volume, a measure of anemia [48]. However there were no significant associations reported with depression [53] or post-concussive syndrome [20].

### Disease pathways

Four studies explored patterns associations between AD pathway PRS and disease-related phenotypes. Each study used sets of SNPs based on previous pathway analyses in AD [6, 73]. Some used only Bonferroni-significant loci [23, 41, 42], whereas others used a threshold-based PRS [32]. Various phenotypes were assessed including: MCI risk [23], MRI phenotypes [23, 41], cognition [42], CSF Aβ and tau [42], Aβ PET [42], and complement markers [32].

Using PRS for the immune response, endocytosis, cholesterol transport, hematopoietic cell lineage, protein ubiquitination, hemostasis, clathrin/AP2 adaptor complex, and protein folding pathway, Ahmad et al. reported the immune response and clathrin/AP2 adaptor complex pathways showed nominal associations with white matter lesions, but this did not withstand correction for multiple testing. The endocytosis risk score was significantly associated with risk of MCI [23]. Darst et al. used PRS for Aβ clearance, cholesterol metabolism, and the immune response. They found no association between cognition and any PRS, even when *APOE* was included [42]. A higher Aβ clearance PRS and cholesterol PRS was associated with lower CSF Aβ_42_, a narrower Aβ_42_/Aβ_40_ ratio, and greater Aβ PET deposition. With APOE excluded, the only significant associations were between the cholesterol PRS and CSF Aβ_42_/Aβ_40_ and the immune response PRS and CSF tau, though not when corrected for multiple comparisons [42].

Two studies focused on the immune response PRS. Corlier et al. found that the immune response PRS was significantly associated with an overall measure of cortical thinning [41]. Morgan et al reported that clusterin, C1 inhibitor, and C-reactive protein all showed nominal association with the inflammation-specific PRS. Plasma clusterin levels were associated with the overall AD PRS [32].

### Study quality

Overall, the articles had clear research questions and used adequate methodology. Some studies used small sample sizes [32, 49, 52, 65] and many studies failed to describe sample ascertainment clearly. They used standard outcome measures. Of those looking at AD prediction, all but two [14, 22] reporting using NINDS-ADRDA diagnostic criteria for AD. Most studies weighted PRS by effect size or odds ratio, although in some studies this was not clearly described [14, 22]. Some studies had some overlap between training and validation datasets which may have inflated their results. Most studies attempted to assess the contribution of *APOE* by either excluding it from the PRS or including it as a co-variate. Some studies included cohorts of non-European ancestry [5, 22, 39, 44, 45]. These studies acknowledged that: 1) they may have had insufficient power in their non-European samples or 2) PRS based on GWAS conducted in European populations may not capture AD genetic risk among those of non-European descent.

## DISCUSSION

This paper systematically reviews how AD PRS are associated with a range of phenotypes and outcomes. Other papers have covered PRS methodology [3] and some have reviewed the use of PRS in AD prediction alone [74].

Since the advent of large-scale genetics consortia such as the International Genomics of Alzheimer’s Project (IGAP), our understanding of the genetic underpinnings of AD has rapidly expanded. GWAS have resulted in the identification of over 20 novel genetic risk loci in addition to *APOE* *ɛ*4 [2, 66]. Most of these SNPs only increase AD risk incrementally. Therefore, combining SNPs into PRS has proved an important strategy for studying their effects. Some of the studies included in this review used only the most significant loci in their PRS. However, more recent studies used liberal threshold-based PRS computed from thousands of AD risk loci.

### PRS in disease prediction

AD PRS have demonstrated strong predictive ability. Conservative PRS, including only genome-wide significant SNPs, have achieved reasonable prediction accuracy (AUC range: 57–72%) [4, 5, 12, 25, 69]. Threshold-based PRS, including many more SNPs, have proved superior to both conservative PRS and to *APOE* alone (AUC 75%) [4]. Prediction accuracy is even greater using a threshold-based PRS in histologically confirmed cases and controls (AUC 84%) [17]. The findings for MCI conversion prediction are more mixed. Of the three studies reporting negative results, two had relatively low power [11, 29]. Almost all the studies exploring PRS prediction accuracy report that there is some overlap between cases and controls at high polygenic risk. Moreover, in the absence of therapeutic consequences, the clinical utility of these findings remains limited.

### Associations between AD PRS, phenotypes, and biomarkers

Overall, the evidence from cross-sectional, case-control and longitudinal cohort studies pointed towards an association between PRS and a range of AD-related phenotypes. Of these, cognition has been the most widely investigated. While the methodology and samples were diverse, the vast majority of studies reported significant associations [11, 26, 31, 33, 36, 40, 44, 45, 50, 52, 54, 56, 60, 63, 64, 68, 69]. Of the negative studies, one used a threshold-based PRS [59] and another used a PRS including 15 Bonferroni-significant risk SNPs [39] but both excluded *APOE* entirely. The other two negative studies both used samples of young adults [51, 57], suggesting that cognitive changes related to AD genetic risk may not manifest until later in life.

There was consistent evidence to support an association between AD PRS and changes in brain structure, particularly in decreased hippocampal volume [30, 33, 40, 51, 61, 72] and reduced cortical thickness [34, 41, 56, 57, 61]. This was reported even in samples of young adults [40, 51], suggesting that AD risk may manifest in brain structure decades before the onset of disease. These studies provide also found that the threshold based PRS yielded better results. For example, Mormino et al. found an association between a threshold PRS and hippocampal volume that was not present when only genome-wide significant SNPs were used [33].

There were mixed findings for amyloid and tau biomarkers. Of those studies exploring CSF, PET, or histology biomarkers, all but one reported statistically significant associations. However, findings were not consistent across biomarkers. For example, one study reported an association between CSF tau and phosphorylated tau but not Aβ [9], whereas another study found the reverse [10]. Another study reported a significant association with Aβ PET but not with CSF Aβ or tau [33]. However, studies with postmortem samples did find evidence of association between AD PRS and soluble Aβ_42_ levels, *λ*-secretase activity [10], neuritic amyloid plaques and neurofibrillary tangles [60]. PRS for other neuropsychiatric disorders were not associated [60]. Moreover, AD PRS was not associated with amyloid accumulation in normal pressure hydrocephalus [8]. This suggests that the genetic foundations of amyloid deposition in other conditions may be distinct from those in AD. In addition, there was no evidence for pleiotropy between AD and depression [53].

### PRS in disease pathways

GWAS have resulted in the identification of novel genetic risk loci in addition to *APOE* *ɛ*4,[2, 66] which have been associated with a range of biological pathways including lipid metabolism, immune response, and synaptic processes [6, 73]. AD is heterogeneous and multifactorial. Polygenic profiling can allow individual molecular sub-classification, by identifying the pathways enriched for risk alleles for an individual. Four of the most recent studies included in this review took this approach, suggesting that the field is moving in this direction. They found some evidence for association between pathway-specific polygenic scores and MCI risk [23], cognition [42], brain structure [23, 41], CSF biomarkers [42], Aβ PET [42], and serum complement markers [32]. The variance that each of these pathways explains is small [42]. This will probably increase as discovery sample sizes increase [75], but will be restricted as PRS do not capture the contributions of copy number variant or rare SNPs.

Pathway-specific polygenic profiling could enable personalized treatment of each individual with AD. This could allow entrants to clinical trials and biomarker studies to be stratified based on evidence of involvement of specific disease pathways. Moreover, if polygenic risk profiles can give prognostic information, they may aid decision making for individuals and clinicians. For example, a high PRS has been associated with a more accelerated progression from MCI to AD [11].

### Strengths and limitations

We used a systematic and comprehensive search strategy to avoid missing eligible studies. However, we were not able to include studies that were not in English-language journals. Another strength is that articles were not limited to a particular sampling framework or research design (e.g., longitudinal studies or clinical samples), or to European ancestry samples. We also included studies investigating broad ranges of outcomes which enhanced our ability to assess how AD polygenic risk is manifest. However, results were not reported consistently across studies, meaning only a narrative review was feasible, and we were not able to assess for publication bias.

We identified a number of limitations in the studies included in this review. In order to conduct a polygenic score analysis, two completely independent datasets are required. Any overlap in the datasets will inflate the associations found. Some studies appeared to use sub-samples of the discovery sample as target samples and not all attempted to account for this. A number of studies used small sample sizes. Authors often did not provide a clear description of sample ascertainment, making it harder to put their findings into the context of the wider literature. Standardized effect estimates or confidence intervals were also often omitted, which are required to compare effect sizes across studies. We have previously proposed a reporting framework for studies which might assist future researchers who synthesize data across such studies [76].

Some studies explored similar phenotypes in comparable samples but reported different results. Heterogeneity may stem from the PRS or the study design. Regarding the PRS, the exact list of SNPs is likely to differ between studies. Some researchers selected SNPs that reached genome-wide significance, and others used a *p*-value threshold approach, a key distinction. With threshold based PRS, experimenters exclude SNPs with low imputation quality scores. These vary depending on the array, imputation platform and pre- and post-imputation quality control steps. In addition, even small differences in population genetics may lead to distinctive linkage disequilibrium (LD) structure and allele frequencies [77]. Pruning, an essential part of PRS calculation, relies on LD structure to retain SNPs that are most associated with a trait while removing others that are closely linked. Where LD structure diverges, alternative SNPs will be selected. Furthermore, in disease pathway PRS, the gene sets are determined by the databases used to define the pathways. Regarding study design, there are other potential causes of heterogeneity. There may be discrepancies in how phenotypes are defined or measured, and different approaches to data analysis. Finally, there are possible sources of bias. For example, disease prediction studies using PRS can be affected by selection bias. If the target dataset is enriched for AD or MCI cases, this will affect the prediction accuracy.

### Conclusions

PRS approach is an important approach used for capturing the contribution of genome wide common variation of complex diseases. To the best of our knowledge, this is the first review attempting to collate information on how the use of the PRS approach has informed our understanding of a variety of phenotypes associated with AD genetic risk. PRS can predict AD and are associated with cognitive impairment. There is also evidence of association between AD PRS and other phenotypes relevant to neurodegeneration. The associations between pathway specific PRS and phenotypic changes may allow us to define the biology of the disease in individuals, heralding precision medicine in AD. However, longitudinal cohort studies are required to test the ability of PRS to delineate pathway-specific disease activity. In the absence of therapeutic consequences, the clinical utility of PRS is limited.

## Supplementary Material

Supplementary Tables 1 and 2

Supplementary Table 3

Supplementary Table 4

## References

[1] Gatz M , Pedersen NL , Berg S , Johansson B , Johansson K , Mortimer JA , Posner SF , Viitanen M , Winblad B , Ahlbom A (1997) Heritability for Alzheimer’s disease: The study of dementia in Swedish twins. J Gerontol A Biol Sci Med Sci 52, M117–M125. 9060980 10.1093/gerona/52a.2.m117

[2] Kunkle BW , Grenier-Boley B , Sims R , Bis JC , Damotte V , Naj AC , Boland A , Vronskaya M , van der Lee SJ , Amlie-Wolf A , Bellenguez C , Frizatti A , Chouraki V , Martin ER , Sleegers K , Badarinarayan N , Jakobsdottir J , Hamilton-Nelson KL , Moreno-Grau S , Olaso R , Raybould R , Chen Y , Kuzma AB , Hiltunen M , Morgan T , Ahmad S , Vardarajan BN , Epelbaum J , Hoffmann P , Boada M , Beecham GW , Garnier J-G , Harold D , Fitzpatrick AL , Valladares O , Moutet M-L , Gerrish A , Smith AV , Qu L , Bacq D , Denning N , Jian X , Zhao Y , Del Zompo M , Fox NC , Choi S-H , Mateo I , Hughes JT , Adams HH , Malamon J , Sanchez-Garcia F , Patel Y , Brody JA , Dombroski BA , Naranjo MCD , Daniilidou M , Eiriksdottir G , Mukherjee S , Wallon D , Uphill J , Aspelund T , Cantwell LB , Garzia F , Galimberti D , Hofer E , Butkiewicz M , Fin B , Scarpini E , Sarnowski C , Bush WS , Meslage S , Kornhuber J , White CC , Song Y , Barber RC , Engelborghs S , Sordon S , Voijnovic D , Adams PM , Vandenberghe R , Mayhaus M , Cupples LA , Albert MS , De Deyn PP , Gu W , Himali JJ , Beekly D , Squassina A , Hartmann AM , Orellana A , Blacker D , Rodriguez-Rodriguez E , Lovestone S , Garcia ME , Doody RS , Munoz-Fernadez C , Sussams R , Lin H , Fairchild TJ , Benito YA , Holmes C , Karamujić-Čomić H , Frosch MP , Thonberg H , Maier W , Roschupkin G , Ghetti B , Giedraitis V , Kawalia A , Li S , Huebinger RM , Kilander L , Moebus S , Hernández I , Kamboh MI , Brundin R , Turton J , Yang Q , Katz MJ , Concari L , Lord J , Beiser AS , Keene CD , Helisalmi S , Kloszewska I , Kukull WA , Koivisto AM , Lynch A , Tarraga L , Larson EB , Haapasalo A , Lawlor B , Mosley TH , Lipton RB , Solfrizzi V , Gill M , Longstreth WT , Montine TJ , Frisardi V , Diez-Fairen M , Rivadeneira F , Petersen RC , Deramecourt V , Alvarez I , Salani F , Ciaramella A , Boerwinkle E , Reiman EM , Fievet N , Rotter JI , Reisch JS , Hanon O , Cupidi C , Andre Uitterlinden AG , Royall DR , Dufouil C , Maletta RG , de Rojas I , Sano M , Brice A , Cecchetti R , George-Hyslop PS , Ritchie K , Tsolaki M , Tsuang DW , Dubois B , Craig D , Wu C-K , Soininen H , Avramidou D , Albin RL , Fratiglioni L , Germanou A , Apostolova LG , Keller L , Koutroumani M , Arnold SE , Panza F , Gkatzima O , Asthana S , Hannequin D , Whitehead P , Atwood CS , Caffarra P , Hampel H , Quintela I , Carracedo Á , Lannfelt L , Rubinsztein DC , Barnes LL , Pasquier F , Frölich L , Barral S , McGuinness B , Beach TG , Johnston JA , Becker JT , Passmore P , Bigio EH , Schott JM , Bird TD , Warren JD , Boeve BF , Lupton MK , Bowen JD , Proitsi P , Boxer A , Powell JF , Burke JR , Kauwe JSK , Burns JM , Mancuso M , Buxbaum JD , Bonuccelli U , Cairns NJ , McQuillin A , Cao C , Livingston G , Carlson CS , Bass NJ , Carlsson CM , Hardy J , Carney RM , Bras J , Carrasquillo MM , Guerreiro R , Allen M , Chui HC , Fisher E , Masullo C , Crocco EA , DeCarli C , Bisceglio G , Dick M , Ma L , Duara R , Graff-Radford NR , Evans DA , Hodges A , Faber KM , Scherer M , Fallon KB , Riemenschneider M , Fardo DW , Heun R , Farlow MR , Kölsch H , Ferris S , Leber M , Foroud TM , Heuser I , Galasko DR , Giegling I , Gearing M , Hüll M , Geschwind DH , Gilbert JR , Morris J , Green RC , Mayo K , Growdon JH , Feulner T , Hamilton RL , Harrell LE , Drichel D , Honig LS , Cushion TD , Huentelman MJ , Hollingworth P , Hulette CM , Hyman BT , Marshall R , Jarvik GP , Meggy A , Abner E , Menzies GE , Jin L-W , Leonenko G , Real LM , Jun GR , Baldwin CT , Grozeva D , Karydas A , Russo G , Kaye JA , Kim R , Jessen F , Kowall NW , Vellas B , Kramer JH , Vardy E , LaFerla FM , Jöckel K-H , Lah JJ , Dichgans M , Leverenz JB , Mann D , Levey AI , Pickering-Brown S , Lieberman AP , Klopp N , Lunetta KL , Wichmann H-E , Lyketsos CG , Morgan K , Marson DC , Brown K , Martiniuk F , Medway C , Mash DC , Nöthen MM , Masliah E , Hooper NM , McCormick WC , Daniele A , McCurry SM , Bayer A , McDavid AN , Gallacher J , McKee AC , van den Bussche H , Mesulam M , Brayne C , Miller BL , Riedel-Heller S , Miller CA , Miller JW , Al-Chalabi A , Morris JC , Shaw CE , Myers AJ , Wiltfang J , O’Bryant S , Olichney JM , Alvarez V , Parisi JE , Singleton AB , Paulson HL , Collinge J , Perry WR , Mead S , Peskind E , Cribbs DH , Rossor M , Pierce A , Ryan NS , Poon WW , Nacmias B , Potter H , Sorbi S , Quinn JF , Sacchinelli E , Raj A , Spalletta G , Raskind M , Caltagirone C , Bossú P , Orfei MD , Reisberg B , Clarke R , Reitz C , Smith AD , Ringman JM , Warden D , Roberson ED , Wilcock G , Rogaeva E , Bruni AC , Rosen HJ , Gallo M , Rosenberg RN , Ben-Shlomo Y , Sager MA , Mecocci P , Saykin AJ , Pastor P , Cuccaro ML , Vance JM , Schneider JA , Schneider LS , Slifer S , Seeley WW , Smith AG , Sonnen JA , Spina S , Stern RA , Swerdlow RH , Tang M , Tanzi RE , Trojanowski JQ , Troncoso JC , Van Deerlin VM , Van Eldik LJ , Vinters HV , Vonsattel JP , Weintraub S , Welsh-Bohmer KA , Wilhelmsen KC , Williamson J , Wingo TS , Woltjer RL , Wright CB , Yu C-E , Yu L , Saba Y , Pilotto A , Bullido MJ , Peters O , Crane PK , Bennett D , Bosco P , Coto E , Boccardi V , De Jager PL , Lleo A , Warner N , Lopez OL , Ingelsson M , Deloukas P , Cruchaga C , Graff C , Gwilliam R , Fornage M , Goate AM , Sanchez-Juan P , Kehoe PG , Amin N , Ertekin-Taner N , Berr C , Debette S , Love S , Launer LJ , Younkin SG , Dartigues J-F , Corcoran C , Ikram MA , Dickson DW , Nicolas G , Campion D , Tschanz J , Schmidt H , Hakonarson H , Clarimon J , Munger R , Schmidt R , Farrer LA , Van Broeckhoven C , C. O’Donovan M , DeStefano AL , Jones L , Haines JL , Deleuze J-F , Owen MJ , Gudnason V , Mayeux R , Escott-Price V , Psaty BM , Ramirez A , Wang L-S , Ruiz A , van Duijn CM , Holmans PA , Seshadri S , Williams J , Amouyel P , Schellenberg GD , Lambert J-C , Pericak-Vance MA (2019) Genetic meta-analysis of diagnosed Alzheimer’s disease identifies new risk loci and implicates Aβ, tau, immunity and lipid processing. Nat Genet 51, 414–430. 30820047 10.1038/s41588-019-0358-2 PMC6463297

[3] Wray NR , Lee SH , Mehta D , Vinkhuyzen AAE , Dudbridge F , Middeldorp CM (2014) Research Review: Polygenic methods and their application to psychiatric traits. J Child Psychol Psychiatry 55, 1068–1087. 25132410 10.1111/jcpp.12295

[4] Escott-Price V , Sims R , Bannister C , Harold D , Vronskaya M , Majounie E , Badarinarayan N , Morgan K , Passmore P , Holmes C , Powell J , Brayne C , Gill M , Mead S , Goate A , Cruchaga C , Lambert J-C , van Duijn C , Maier W , Ramirez A , Holmans P , Jones L , Hardy J , Seshadri S , Schellenberg GD , Amouyel P , Williams J (2015) Common polygenic variation enhances risk prediction for Alzheimer’s disease. Brain 138, 3673–3684. 26490334 10.1093/brain/awv268 PMC5006219

[5] Tosto G , Bird TD , Tsuang D , Bennett DA , Boeve BF , Cruchaga C , Faber K , Foroud TM , Farlow M , Goate AM , Bertlesen S , Graff-Radford NR , Medrano M , Lantigua R , Manly J , Ottman R , Rosenberg R , Schaid DJ , Schupf N , Stern Y , Sweet RA , Mayeux R (2017) Polygenic risk scores in familial Alzheimer disease. Neurology 88, 1180–1186. 28213371 10.1212/WNL.0000000000003734 PMC5373783

[6] Jones L , Holmans PA , Hamshere ML , Harold D , Moskvina V , Ivanov D , Pocklington A , Abraham R , Hollingworth P , Sims R , Gerrish A , Pahwa JS , Jones N , Stretton A , Morgan AR , Lovestone S , Powell J , Proitsi P , Lupton MK , Brayne C , Rubinsztein DC , Gill M , Lawlor B , Lynch A , Morgan K , Brown KS , Passmore PA , Craig D , McGuinness B , Todd S , Holmes C , Mann D , Smith AD , Love S , Kehoe PG , Mead S , Fox N , Rossor M , Collinge J , Maier W , Jessen F , Schürmann B , Heun R , Kölsch H , van den Bussche H , Heuser I , Peters O , Kornhuber J , Wiltfang J , Dichgans M , Frölich L , Hampel H , Hüll M , Rujescu D , Goate AM , Kauwe JSK , Cruchaga C , Nowotny P , Morris JC , Mayo K , Livingston G , Bass NJ , Gurling H , McQuillin A , Gwilliam R , Deloukas P , Al-Chalabi A , Shaw CE , Singleton AB , Guerreiro R , Mühleisen TW , Nöthen MM , Moebus S , Jöckel K-H , Klopp N , Wichmann H-E , Rüther E , Carrasquillo MM , Pankratz VS , Younkin SG , Hardy J , O’Donovan MC , Owen MJ , Williams J (2010) Genetic evidence implicates the immune system and cholesterol metabolism in the aetiology of Alzheimer’s disease. PLoS One 5, e13950. 21085570 10.1371/journal.pone.0013950 PMC2981526

[7] Moher D , Liberati A , Tetzlaff J , Altman DG (2009) Preferred reporting items for systematic reviews and meta-analyses: The PRISMA Statement. Ann Intern Med 151, 264–269. 19622511 10.7326/0003-4819-151-4-200908180-00135

[8] Laiterä T , Paananen J , Helisalmi S , Sarajärvi T , Huovinen J , Laitinen M , Rauramaa T , Alafuzoff I , Remes AM , Soininen H , Haapasalo A , Jääskeläinen JE , Leinonen V , Hiltunen M (2016) Effects of Alzheimer’s disease-associated risk loci on amyloid-β accumulation in the brain of idiopathic normal pressure hydrocephalus patients. J Alzheimers Dis 55, 995–1003. 10.3233/JAD-160554 27802227

[9] Louwersheimer E , Wolfsgruber S , Espinosa A , Lacour A , Heilmann-Heimbach S , Alegret M , Hernández I , Rosende-Roca M , Tárraga L , Boada M , Kornhuber J , Peters O , Frölich L , Hüll M , Rüther E , Wiltfang J , Scherer M , Riedel-Heller S , Jessen F , Nöthen MM , Maier W , Koene T , Scheltens P , Holstege H , Wagner M , Ruiz A , van der Flier WM , Becker T , Ramirez A (2016) Alzheimer’s disease risk variants modulate endophenotypes in mild cognitive impairment. Alzheimers Dement 12, 872–881. 26921674 10.1016/j.jalz.2016.01.006

[10] Martiskainen H , Helisalmi S , Viswanathan J , Kurki M , Hall A , Herukka SK , Sarajärvi T , Natunen T , Kurkinen KMA , Huovinen J , Mäkinen P , Laitinen M , Koivisto AM , Mattila KM , Lehtimäki T , Remes AM , Leinonen V , Haapasalo A , Soininen H , Hiltunen M (2015) Effects of Alzheimer’s disease-associated risk loci on cerebrospinal fluid biomarkers and disease progression: A polygenic risk score approach. J Alzheimers Dis 43, 565–573. 25096612 10.3233/JAD-140777

[11] Rodríguez-Rodríguez E , Sánchez-Juan P , Vázquez-Higuera JL , Mateo I , Pozueta A , Berciano J , Cervantes S , Alcolea D , Martínez-Lage P , Clarimón J , Lleó A , Pastor P , Combarros O (2013) Genetic risk score predicting accelerated progression from mild cognitive impairment to Alzheimer’s disease. J Neural Transm 120, 807–812. 23180304 10.1007/s00702-012-0920-x

[12] Sleegers K , Bettens K , De Roeck A , Van Cauwenberghe C , Cuyvers E , Verheijen J , Struyfs H , Van Dongen J , Vermeulen S , Engelborghs S , Vandenbulcke M , Vandenberghe R , De Deyn PP , Van Broeckhoven C (2015) A 22-single nucleotide polymorphism Alzheimer’s disease risk score correlates with family history, onset age, and cerebrospinal fluid Aβ42. Alzheimers Dement 11, 1452–1460. 26086184 10.1016/j.jalz.2015.02.013

[13] Su F , Shu H , Ye Q , Xie C , Yuan B , Zhang Z , Bai F (2017) Integration of multilocus genetic risk into the default mode network longitudinal trajectory during the Alzheimer’s disease process. J Alzheimers Dis 56, 491–507. 28035927 10.3233/JAD-160787

[14] Yokoyama JS , Lee AKL , Takada LT , Busovaca E , Bonham LW , Chao SZ , Tse M , He J , Schwarz CG , Carmichael OT , Matthews BR , Karydas A , Weiner MW , Coppola G , DeCarli CS , Miller BL , Rosen HJ (2015) Apolipoprotein epsilon 4 is associated with lower brain volume in cognitively normal Chinese but not white older adults. PLoS One 10, e0118338. 25738563 10.1371/journal.pone.0118338 PMC4349764

[15] Chaudhury S , Patel T , Barber IS , Guetta-Baranes T , Brookes KJ , Chappell S , Turton J , Guerreiro R , Bras J , Hernandez D , Singleton A , Hardy J , Mann D , Morgan K , Passmore P , Craig D , Johnston J , McGuinness B , Todd S , Heun R , Kölsch H , Kehoe PG , Vardy ERLC , Hooper NM , Pickering-Brown S , Snowden J , Richardson A , Jones M , Neary D , Harris J , Lowe J , Smith AD , Wilcock G , Warden D , Holmes C (2018) Polygenic risk score in postmortem diagnosed sporadic early-onset Alzheimer’s disease. Neurobiol Aging 62, 244.e1–244.e8. 10.1016/j.neurobiolaging.2017.09.035 PMC5995122 29103623

[16] Escott-Price V , Sims R , Harold D , Vronskaya M , Holmans P , Williams J (2015) Using polygenic risk score to predict Alzheimer’s disease. Alzheimers Dement 11, P872.

[17] Escott-Price V , Myers AJ , Huentelman M , Hardy J (2017) Polygenic risk score analysis of pathologically confirmed Alzheimer disease. Ann Neurol 82, 311–314. 28727176 10.1002/ana.24999 PMC5599118

[18] Adams HHH , De Bruijn RFAG , Hofman A , Uitterlinden AG , Van Duijn CM , Vernooij MW , Koudstaal PJ , Ikram MA (2015) Genetic risk of neurodegenerative diseases is associated with mild cognitive impairment and conversion to dementia. Alzheimers Dement 11, 1277–1285. 25916564 10.1016/j.jalz.2014.12.008

[19] Patel T , Brookes KJ , Turton J , Chaudhury S , Guetta-Baranes T , Guerreiro R , Bras J , Hernandez D , Singleton A , Francis PT , Hardy J , Morgan K (2018) Whole-exome sequencing of the BDR cohort: Evidence to support the role of thegene in Alzheimer’s disease. Neuropathol Appl Neurobiol 44, 506–521. 29181857 10.1111/nan.12452 PMC6005734

[20] Polimanti R , Chen C-Y , Ursano RJ , Heeringa SG , Jain S , Kessler RC , Nock MK , Smoller JW , Sun X , Gelernter J , Stein MB (2017) Cross-phenotype polygenic risk score analysis of persistent post-concussive symptoms in U.S. Army soldiers with deployment-acquired traumatic brain injury. J Neurotrauma 34, 781–789. 27439997 10.1089/neu.2016.4550 PMC5314978

[21] Voyle N , Patel H , Folarin A , Newhouse S , Johnston C , Visser PJ , Dobson RJB , Kiddle SJ , EDAR and DESCRIPA study groups and the Alzheimer’s Disease Neuroimaging Initiative (2016) Genetic risk as a marker of amyloid-β and tau burden in cerebrospinal fluid. J Alzheimers Dis 55, 1417–1427. 10.3233/JAD-160707 PMC5181674 27834776

[22] Xiao Q , Liu Z-J , Tao S , Sun Y-M , Jiang D , Li H-L , Chen H , Liu X , Lapin B , Wang C-H , Zheng SL , Xu J , Wu Z-Y (2015) Risk prediction for sporadic Alzheimer’s disease using genetic risk score in the Han Chinese population. Oncotarget 6, 36955–36964. 26543236 10.18632/oncotarget.6271 PMC4741908

[23] Ahmad S , Bannister C , van der Lee SJ , Vojinovic D , Adams HHH , Ramirez A , Escott-Price V , Sims R , Baker E , Williams J , Holmans P , Vernooij MW , Ikram MA , Amin N , van Duijn CM (2018) Disentangling the biological pathways involved in early features of Alzheimer’s disease in the Rotterdam Study. Alzheimers Dement 14, 848–857. 29494809 10.1016/j.jalz.2018.01.005

[24] Chouraki V , De Bruijn RFAG , Chapuis J , Bis JC , Reitz C , Schraen S , Ibrahim-Verbaas CA , Grenier-Boley B , Delay C , Rogers R , Demiautte F , Mounier A , Fitzpatrick AL , Berr C , Dartigues JF , Uitterlinden AG , Hofman A , Breteler M , Becker JT , Lathrop M , Schupf N , Alpérovitch A , Mayeux R , Van Duijn CM , Buée L , Amouyel P , Lopez OL , Ikram MA , Tzourio C , Lambert JC (2014) A genome-wide association meta-analysis of plasma Aβ peptides concentrations in the elderly. Mol Psychiatry 19, 1326–1335. 24535457 10.1038/mp.2013.185 PMC4418478

[25] Cruchaga C , Del-Aguila JL , Saef B , Black K , Fernandez MV , Budde J , Ibanez L , Deming Y , Kapoor M , Tosto G , Mayeux RP , Holtzman DM , Fagan AM , Morris JC , Bateman RJ , Goate AM , Dominantly Inherited Alzheimer Network (DIAN); Disease Neuroimaging Initiative (ADNI); NIA-LOAD family study, Harari O (2018) Polygenic risk score of sporadic late-onset Alzheimer’s disease reveals a shared architecture with the familial and early-onset forms. Alzheimers Dement 14, 205–214. 28943286 10.1016/j.jalz.2017.08.013 PMC5803427

[26] Del-Aguila J , Fernández M , Schindler S , Ibanez L , Deming Y , Ma S , Saef B , Black K , Budde J , Norton J , Chasse R , Harari O , Goate A , Xiong C , Morris J , Cruchaga C (2018) Assessment of the genetic architecture of Alzheimer’s Disease risk in rate of memory decline. J Alzheimers Dis 62, 745–756. 29480181 10.3233/JAD-170834 PMC5989565

[27] Dubé JB , Johansen CT , Robinson JF , Lindsay J , Hachinski V , Hegele RA (2013) Genetic determinants of “cognitive impairment, no dementia.” J Alzheimers Dis 33, 831–840. 23042215 10.3233/JAD-2012-121477

[28] Hohman TJ , Dumitrescu L , Oksol A , Wagener M , Gifford KA , Jefferson AL , Alzheimer’s Disease Neuroimaging Initiative (2017) APOE allele frequencies in suspected non-amyloid pathophysiology (SNAP) and the prodromal stages of Alzheimer’s Disease. PLoS One 12, e0188501. 29190651 10.1371/journal.pone.0188501 PMC5708777

[29] Lacour A , Espinosa A , Louwersheimer E , Heilmann S , Hernández I , Wolfsgruber S , Fernández V , Wagner H , Rosende-Roca M , Mauleón A , Moreno-Grau S , Vargas L , Pijnenburg YAL , Koene T , Rodríguez-Gómez O , Ortega G , Ruiz S , Holstege H , Sotolongo-Grau O , Kornhuber J , Peters O , Frölich L , Hüll M , Rüther E , Wiltfang J , Scherer M , Riedel-Heller S , Alegret M , Nöthen MM , Scheltens P , Wagner M , Tárraga L , Jessen F , Boada M , Maier W , Van Der Flier WM , Becker T , Ramirez A , Ruiz A (2017) Genome-wide significant risk factors for Alzheimer’s disease: Role in progression to dementia due to Alzheimer’s disease among subjects with mild cognitive impairment. Mol Psychiatry 22, 153–160. 26976043 10.1038/mp.2016.18 PMC5414086

[30] Lupton MK , Strike L , Hansell NK , Wen W , Mather KA , Armstrong NJ , Thalamuthu A , McMahon KL , de Zubicaray GI , Assareh AA , Simmons A , Proitsi P , Powell JF , Montgomery GW , Hibar DP , Westman E , Tsolaki M , Kloszewska I , Soininen H , Mecocci P , Velas B , Lovestone S , Brodaty H , Ames D , Trollor JN , Martin NG , Thompson PM , Sachdev PS , Wright MJ (2016) The effect of increased genetic risk for Alzheimer’s disease on hippocampal and amygdala volume. Neurobiol Aging 40, 68–77. 26973105 10.1016/j.neurobiolaging.2015.12.023 PMC4883003

[31] Marioni RE , Campbell A , Hagenaars SP , Nagy R , Amador C , Hayward C , Porteous DJ , Visscher PM , Deary IJ (2017) Genetic stratification to identify risk groups for Alzheimer’s disease. J Alzheimers Dis 57, 275–283. 28222519 10.3233/JAD-161070 PMC5345653

[32] Morgan AR , Touchard S , O’Hagan C , Sims R , Majounie E , Escott-Price V , Jones L , Williams J , Morgan BP (2017) The correlation between inflammatory biomarkers and polygenic risk score in Alzheimer’s disease. J Alzheimers Dis 56, 25–36. 27911318 10.3233/JAD-160889

[33] Mormino EC , Sperling RA , Holmes AJ , Buckner RL , De Jager PL , Smoller JW , Sabuncu MR , Alzheimer’s Disease Neuroimaging Initiative (2016) Polygenic risk of Alzheimer disease is associated with early- and late-life processes. Neurology 87, 481–488. 27385740 10.1212/WNL.0000000000002922 PMC4970660

[34] Sabuncu MR , Buckner RL , Smoller JW , Lee PH , Fischl B , Sperling RA (2012) The association between a polygenic Alzheimer score and cortical thickness in clinically normal subjects. Cereb Cortex 22, 2653–2661. 22169231 10.1093/cercor/bhr348 PMC3464416

[35] Tan CH , Fan CC , Mormino EC , Sugrue LP , Broce IJ , Hess CP , Dillon WP , Bonham LW , Yokoyama JS , Karch CM , Brewer JB , Rabinovici GD , Miller BL , Schellenberg GD , Kauppi K , Feldman HA , Holland D , McEvoy LK , Hyman BT , Bennett DA , Andreassen OA , Dale AM , Desikan RS (2018) Polygenic hazard score: An enrichment marker for Alzheimer’s associated amyloid and tau deposition. Acta Neuropathol 135, 85–93. 29177679 10.1007/s00401-017-1789-4 PMC5758038

[36] Andrews SJ , Das D , Anstey KJ , Easteal S (2017) Late onset Alzheimer’s disease risk variants in cognitive decline: The PATH Through Life Study. J Alzheimers Dis 57, 423–436. 28269768 10.3233/JAD-160774

[37] Andrews SJ , Eramudugolla R , Velez JI , Cherbuin N , Easteal S , Anstey KJ (2017) Validating the role of the Australian National University Alzheimer’s Disease Risk Index (ANU-ADRI) and a genetic risk score in progression to cognitive impairment in a population-based cohort of older adults followed for 12 years. Alzheimers Res Ther 9, 16. 28259165 10.1186/s13195-017-0240-3 PMC5336661

[38] Andrews SJ , Das D , Cherbuin N , Anstey KJ , Easteal S (2016) Association of genetic risk factors with cognitive decline: The PATH through life project. Neurobiol Aging 41, 150–158. 27103528 10.1016/j.neurobiolaging.2016.02.016

[39] Bressler J , Mosley TH , Penman A , Gottesman RF , Windham BG , Knopman DS , Wruck LM , Boerwinkle E (2017) Genetic variants associated with risk of Alzheimer’s disease contribute to cognitive change in midlife: The Atherosclerosis Risk in Communities Study. Am J Med Genet Part B Neuropsychiatr Genet 174, 269–282. 10.1002/ajmg.b.32509 PMC5935000 27781389

[40] Axelrud LK , Santoro ML , Pine DS , Talarico F , Gadelha A , Manfro GG , Pan PM , Jackowski A , Picon F , Brietzke E , Grassi-Oliveira R , Bressan RA , Miguel EC , Rohde LA , Hakonarson H , Pausova Z , Belangero S , Paus T , Salum GA (2018) Polygenic risk score for Alzheimer’s disease: Implications for memory performance and hippocampal volumes in early life. Am J Psychiatry 175, 555–563. 29495896 10.1176/appi.ajp.2017.17050529 PMC6372950

[41] Corlier F , Hafzalla G , Faskowitz J , Kuller LH , Becker JT , Lopez OL , Thompson PM , Braskie MN (2018) Systemic inflammation as a predictor of brain aging: Contributions of physical activity, metabolic risk, and genetic risk. Neuroimage 172, 118–129. 29357308 10.1016/j.neuroimage.2017.12.027 PMC5954991

[42] Darst BF , Koscik RL , Racine AM , Oh JM , Krause RA , Carlsson CM , Zetterberg H , Blennow K , Christian BT , Bendlin BB , Okonkwo OC , Hogan KJ , Hermann BP , Sager MA , Asthana S , Johnson SC , Engelman CD (2017) Pathway-specific polygenic risk scores as predictors of amyloid-β deposition and cognitive function in a sample at increased risk for Alzheimer’s disease. J Alzheimers Dis 55, 473–484. 27662287 10.3233/JAD-160195 PMC5123972

[43] Habes M , Janowitz D , Erus G , Toledo JB , Resnick SM , Doshi J , Van der Auwera S , Wittfeld K , Hegenscheid K , Hosten N , Biffar R , Homuth G , Völzke H , Grabe HJ , Hoffmann W , Davatzikos C (2016) Advanced brain aging: Relationship with epidemiologic and genetic risk factors and overlap with Alzheimer disease atrophy patterns. Transl Psychiatry 6, e775. 27045845 10.1038/tp.2016.39 PMC4872397

[44] Marden JR , Mayeda ER , Walter S , Vivot A , Tchetgen Tchetgen EJ , Kawachi I , Glymour MM (2016) Using an Alzheimer disease polygenic risk score to predict memory decline in black and white Americans over 14 years of follow-up. Alzheimer Dis Assoc Disord 30, 195–202. 26756387 10.1097/WAD.0000000000000137 PMC4940299

[45] Marden JR , Walter S , Tchetgen Tchetgen EJ , Kawachi I , Glymour MM (2014) Validation of a polygenic risk score for dementia in black and white individuals. Brain Behav 4, 687–697. 25328845 10.1002/brb3.248 PMC4107377

[46] Papenberg G , Salami A , Persson J , Lindenberger U , Backman L (2015) Genetics and functional imaging: Effects of APOE, BDNF, COMT, and KIBRA in aging. Neuropsychol Rev 25, 47–62. 25666727 10.1007/s11065-015-9279-8

[47] Pilling LC , Atkins JL , Bowman K , Jones SE , Tyrrell J , Beaumont RN , Ruth KS , Tuke MA , Yaghootkar H , Wood AR , Freathy RM , Murray A , Weedon MN , Xue L , Lunetta K , Murabito JM , Harries LW , Robine J-M , Brayne C , Kuchel GA , Ferrucci L , Frayling TM , Melzer D (2016) Human longevity is influenced by many genetic variants: Evidence from 75,000 UK Biobank participants. Aging (Albany NY) 8, 547–560. 27015805 10.18632/aging.100930 PMC4833145

[48] Pilling LC , Atkins JL , Duff MO , Beaumont RN , Jones SE , Tyrrell J , Kuo C-L , Ruth KS , Tuke MA , Yaghootkar H , Wood AR , Murray A , Weedon MN , Harries LW , Kuchel GA , Ferrucci L , Frayling TM , Melzer D (2017) Red blood cell distribution width: Genetic evidence for aging pathways in 116,666 volunteers. PLoS One 12, e0185083. 28957414 10.1371/journal.pone.0185083 PMC5619771

[49] Schultz SA , Boots EA , Darst BF , Zetterberg H , Blennow K , Edwards DF , Koscik RL , Carlsson CM , Gallagher CL , Bendlin BB , Asthana S , Sager MA , Hogan KJ , Hermann BP , Cook DB , Johnson SC , Engelman CD , Okonkwo OC (2017) Cardiorespiratory fitness alters the influence of a polygenic risk score on biomarkers of AD. Neurology 88, 1650–1658. 28341646 10.1212/WNL.0000000000003862 PMC5405766

[50] Verhaaren BFJ , Vernooij MW , Koudstaal PJ , Uitterlinden AG , van Duijn CM , Hofman A , Breteler MMB , Ikram MA (2013) Alzheimer’s disease genes and cognition in the nondemented general population. Biol Psychiatry 73, 429–434. 22592056 10.1016/j.biopsych.2012.04.009

[51] Foley SF , Tansey KE , Caseras X , Lancaster T , Bracht T , Parker G , Hall J , Williams J , Linden D (2016) Multimodal brain imaging reveals structural differences in Alzheimer’s disease polygenic risk carriers: A study in healthy young adults. Biol Psychiatry 81, 154–161. 27157680 10.1016/j.biopsych.2016.02.033 PMC5177726

[52] Wollam ME , Weinstein AM , Saxton JA , Morrow L , Snitz B , Fowler NR , Suever Erickson BL , Roecklein KA , Erickson KI (2015) Genetic risk score predicts late-life cognitive impairment. J Aging Res 2015, 267062. 26366299 10.1155/2015/267062 PMC4561094

[53] Gibson J , Russ TC , Adams MJ , Clarke T-K , Howard DM , Hall LS , Fernandez-Pujals AM , Wigmore EM , Hayward C , Davies G , Murray AD , Smith BH , Porteous DJ , Deary IJ , McIntosh AM (2017) Assessing the presence of shared genetic architecture between Alzheimer’s disease and major depressive disorder using genome-wide association data. Transl Psychiatry 7, e1094. 28418403 10.1038/tp.2017.49 PMC5416691

[54] Hagenaars SP , Harris SE , Davies G , Hill WD , Liewald DCM , Ritchie SJ , Marioni RE , Fawns-Ritchie C , Cullen B , Malik R , METASTROKE Consortium, International Consortium for Blood Pressure GWAS; SpiroMeta Consortium; CHARGE Consortium Pulmonary Group, CHARGE Consortium Aging and Longevity Group, Worrall BB , Sudlow CL , Wardlaw JM , Gallacher J , Pell J , McIntosh AM , Smith DJ , Gale CR , Deary IJ (2016) Shared genetic aetiology between cognitive functions and physical and mental health in UK Biobank (=112 151) and 24 GWAS consortia. Mol Psychiatry 21, 1624–1632. 26809841 10.1038/mp.2015.225 PMC5078856

[55] Hagenaars SP , Harris SE , Davies G , Marioni RE , Liewald DC , Hill WD , Ritchie SJ , Luciano M , Fawns-Ritchie C , Lyall D , Cullen B , Cox SR , Hayward C , Porteous DJ , Evans J , McIntosh AM , Gallacher J , Craddock N , Pell JP , Smith DJ , Gale CR , Deary IJ (2016) Genome-wide association study of cognitive functions and educational attainment in UK Biobank (=112 151). Mol Psychiatry 21, 758–767. 27046643 10.1038/mp.2016.45 PMC4879186

[56] Hayes JP , Logue MW , Sadeh N , Spielberg JM , Verfaellie M , Hayes SM , Reagan A , Salat DH , Wolf EJ , McGlinchey RE , Milberg WP , Stone A , Schichman SA , Miller MW (2017) Mild traumatic brain injury is associated with reduced cortical thickness in those at risk for Alzheimer’s disease. Brain 140, 813–825. 28077398 10.1093/brain/aww344 PMC6075586

[57] Li J , Zhang X , Li A , Liu S , Qin W , Yu C , Liu Y , Liu B , Jiang T (2018) Polygenic risk for Alzheimer’s disease influences precuneal volume in two independent general populations. Neurobiol Aging 64, 116–122. 29358118 10.1016/j.neurobiolaging.2017.12.022

[58] Logue MW , Panizzon MS , Elman JA , Gillespie NA , Hatton SN , Gustavson DE , Andreassen OA , Dale AM , Franz CE , Lyons MJ , Neale MC , Reynolds CA , Tu X , Kremen WS (2019) Use of an Alzheimer’s disease polygenic risk score to identify mild cognitive impairment in adults in their 50s. Mol Psychiatry 24, 421–430. 29487403 10.1038/s41380-018-0030-8 PMC6110977

[59] Harris SE , Davies G , Luciano M , Payton A , Fox HC , Haggarty P , Ollier W , Horan M , Porteous DJ , Starr JM , Whalley LJ , Pendleton N , Deary IJ (2014) Polygenic risk for alzheimer’s disease is not associated with cognitive ability or cognitive aging in non-demented older people. J Alzheimers Dis 39, 565–574. 24246418 10.3233/JAD-131058

[60] Felsky D , Patrick E , Schneider JA , Mostafavi S , Gaiteri C , Patsopoulos N , Bennett DA , De Jager PL (2018) Polygenic analysis of inflammatory disease variants and effects on microglia in the aging brain. Mol Neurodegener 13, 38. 30041668 10.1186/s13024-018-0272-6 PMC6057096

[61] Harrison TM , Bookheimer SY (2016) Neuroimaging genetic risk for Alzheimer’s disease in preclinical individuals: From candidate genes to polygenic approaches. Biol Psychiatry Cogn Neurosci Neuroimaging 1, 14–23. 26858991 10.1016/j.bpsc.2015.09.003 PMC4743051

[62] Hayden KM , Lutz MW , Kuchibhatla M , Germain C , Plassman BL (2015) Effect of APOE and CD33 on cognitive decline. PLoS One 10, e0130419. 26102276 10.1371/journal.pone.0130419 PMC4478019

[63] Sapkota S , Dixon RA (2018) A network of genetic effects on non-demented cognitive aging: Alzheimer’s genetic risk (CLU + CR1 + PICALM) intensifies cognitive aging genetic risk (COMT+BDNF) selectively for APOE *ɛ*4 carriers. J Alzheimers Dis 62, 887–900. 29480189 10.3233/JAD-170909 PMC5830167

[64] Carrasquillo MM , Crook JE , Pedraza O , Thomas CS , Pankratz VS , Allen M , Nguyen T , Malphrus KG , Ma L , Bisceglio GD , Roberts RO , Lucas JA , Smith GE , Ivnik RJ , Machulda MM , Graff-Radford NR , Petersen RC , Younkin SG , Ertekin-Taner N (2015) Late-onset Alzheimer’s risk variants in memory decline, incident mild cognitive impairment, and Alzheimer’s disease. Neurobiol Aging 36, 60–67. 25189118 10.1016/j.neurobiolaging.2014.07.042 PMC4268433

[65] Harrison TM , Mahmood Z , Lau EP , Karacozoff AM , Burggren AC , Small GW , Bookheimer SY (2016) An Alzheimer’s disease genetic risk score predicts longitudinal thinning of hippocampal complex subregions in healthy older adults. eNeuro 3, ENEURO.0098-16.2016. 10.1523/ENEURO.0098-16.2016 PMC4945997 27482534

[66] Lambert J-C , Ibrahim-Verbaas CA , Harold D , Naj AC , Sims R , Bellenguez C , Jun G , DeStefano AL , Bis JC , Beecham GW (2013) Meta-analysis of 74,046 individuals identifies 11 new susceptibility loci for Alzheimer’s disease. Nat Genet 45, 1452–1458. 24162737 10.1038/ng.2802 PMC3896259

[67] Purcell SM , Wray NR , Stone JL , Visscher PM , O’Donovan MC , Sullivan PF , Sklar P , Ruderfer DM , McQuillin A , Morris DW (2009) Common polygenic variation contributes to risk of schizophrenia and bipolar disorder. Nature 460, 748–752. 19571811 10.1038/nature08185 PMC3912837

[68] Papenberg G , Becker N , Ferencz B , Naveh-Benjamin M , Laukka EJ , Bäckman L , Brehmer Y (2017) Dopamine receptor genes modulate associative memory in old age. J Cogn Neurosci 29, 245–253. 27647283 10.1162/jocn_a_01048

[69] Yokoyama JS , Bonham LW , Sears RL , Klein E , Karydas A , Kramer JH , Miller BL , Coppola G (2015) Decision tree analysis of genetic risk for clinically heterogeneous Alzheimer’s disease. BMC Neurol 15, 47. 25880661 10.1186/s12883-015-0304-6 PMC4459447

[70] Chouraki V , Reitz C , Maury F , Bis JC , Bellenguez C , Yu L , Jakobsdottir J , Mukherjee S , Adams HH , Choi SH , Larson EB , Fitzpatrick A , Uitterlinden AG , Jager PL de , Hofman A , Gudnason V , Vardarajan B , Ibrahim-Verbaas C , Lee SJ van der , Lopez O , Dartigues J-F , Berr C , Amouyel P , Bennett DA , Duijn C van , DeStefano AL , Launer LJ , Ikram MA , Crane PK , Lambert J-C , Mayeux R , Seshadri S , International Genomics of Alzheimer’s Project (2016) Evaluation of a genetic risk score to improve risk prediction for Alzheimer’s disease. J Alzheimers Dis 53, 921–932. 27340842 10.3233/JAD-150749 PMC5036102

[71] Biffi A , Anderson CD , Desikan RS , Sabuncu M , Cortellini L , Schmansky N , Salat D , Rosand J (2010) Genetic variation and neuroimaging measures in Alzheimer disease. Arch Neurol 67, 677–685. 20558387 10.1001/archneurol.2010.108 PMC2956757

[72] Hohman TJ , Koran MEI , Thornton-Wells TA , Alzheimer’s Neuroimaging Initiative (2014) Genetic variation modifies risk for neurodegeneration based on biomarker status. Front Aging Neurosci 6, 183. 25140149 10.3389/fnagi.2014.00183 PMC4121544

[73] Holmans P , Jones L (2012) Pathway analysis of IGAP GWAS data implicates endocytosis in the aetiology of late-onset Alzheimer’s disease. Alzheimers Dement 8, P102.

[74] Stocker H , Möllers T , Perna L , Brenner H (2018) The genetic risk of Alzheimer’s disease beyond APOE *ɛ*4: Systematic review of Alzheimer’s genetic risk scores. Transl Psychiatry 8, 166. 30143603 10.1038/s41398-018-0221-8 PMC6109140

[75] Dudbridge F (2013) Power and predictive accuracy of polygenic risk scores. PLoS Genet 9, e1003348. 23555274 10.1371/journal.pgen.1003348 PMC3605113

[76] Mistry S , Harrison JR , Smith DJ , Escott-Price V , Zammit S (2018) The use of polygenic risk scores to identify phenotypes associated with genetic risk of schizophrenia: Systematic review. Schizophr Res 197, 2–8. 29129507 10.1016/j.schres.2017.10.037

[77] Moskvina V , Smith M , Ivanov D , Blackwood D , Stclair D , Hultman C , Toncheva D , Gill M , Corvin A , O’Dushlaine C , Morris DW , Wray NR , Sullivan P , Pato C , Pato MT , Sklar P , Purcell S , Holmans P , O’Donovan MC , Owen MJ , Kirov G (2010) Genetic differences between five european populations. Hum Hered 70, 141–149. 20616560 10.1159/000313854 PMC7077089

