# Supplementary material for: From Polygenic Scores to Precision Medicine in Alzheimer’s Disease: A Systematic Review
Source: J Alzheimers Dis. 2020 Apr 21;74(4):1271–83. doi: 10.3233/JAD-191233 (PMC7242840; doi:10.3233/JAD-191233)
Supplement: Supplementary Tables 1 and 2 [file jad-74-jad191233-s001.pdf]

Supplementary Material

From Polygenic Scores to Precision Medicine in Alzheimer's Disease: A Systematic Review

Supplementary Table 1. Search strategy terms used for searching Embase, Medline via Ovid and PsychINFO.

**Key word**
1. Polygenic risk score.mp
2. Risk profile score.mp
3. Polygenic variation.mp
4. Genome-wide association study/
5. GWAS.mp
6. Gene score.mp
7. Genetic score.mp
8. Allele score.mp
9. Polygenic.mp
10. exp Polymorphism, single nucleotide/
11. SNP score.mp
12. or/1-10
13. exp Alzheimer disease/
14. Alzheimer*.mp
15. or/13-14
16. 12 and 15
17. limit 16 to:<br>- English language<br>- Humans<br>- Yr= "2009-3rd August 2018"

GWAS, genome wide association studies; SNP, single nucleotide polymorphism

Supplementary Table 2. List of data extracted from all studies.

**Details**
• Author
• Year
• Discovery sample
• Target sample
• Outcome measure(s)
• N
• p value thresholds
• Phenotypes/correlates
• β, odds ratio or hazard ratio
• Confidence intervals
• p
• R2 (%)

Supplementary Table 3. Studies examining associations with threshold-based PRS, principle results. (Excel file)

Supplementary Table 4. Studies examining associations with Bonferroni-significant SNP PRS, principle results. (Excel file)
